# Carbazole Alkaloids from *Clausena anisum-olens*: Isolation, Characterization, and Anti-HIV Evaluation

**DOI:** 10.3390/molecules25010099

**Published:** 2019-12-26

**Authors:** Jing-Hua Yang, Xin-Yi Wang, Yi-Ping Zhou, Rong Lu, Chin-Ho Chen, Meng-Han Zhang, Yung-Yi Cheng, Susan L. Morris-Natschke, Kuo-Hsiung Lee, Yun-Song Wang

**Affiliations:** 1 Key Laboratory of Medicinal Chemistry for Natural Resource, Ministry of Education, School of Chemical Science and Technology, Yunnan University, Kunming 650091, China; yangjh@ynu.edu.cn (J.-H.Y.); yangynu@126.com (R.L.); 2 The High School Affiliated to Yunnan Normal University, Kunming 650106, China; wxyyn2019@163.com; 3 School of Pharmaceutical Sciences & Yunnan Key Laboratory of Pharmacology for Natural Products, Kunming Medical University, Kunming 650500, China; zhouypym@foxmail.com; 4 Surgical Science, Department of Surgery, Duke University Medical Center, Durham, NC 27710, USA; chc@duke.edu; 5 Natural Products Research Laboratories, UNC Eshelman School of Pharmacy, University of North Carolina, Chapel Hill, NC 27599, USAyungyi@email.unc.edu (Y.-Y.C.); susan_natschke@unc.edu (S.L.M.-N.); 6 Chinese Medicine Research and Development Center, China Medical University and Hospital, Taichung 40402, Taiwan

**Keywords:** Rutaceae, *Clausena anisum-olens*, carbazole alkaloids, anti-HIV

## Abstract

Two new carbazole alkaloids (**1**,**2**) and six known carbazole alkaloids (**3**–**8**) were isolated from *Clausena anisum-olens*. Their structures were elucidated based on extensive spectroscopic analysis. All isolated compounds (**1**–**8**) were evaluated for their anti-HIV effects on virus replication in MT-4 lymphocytes infected by HIV-1_NL4-3_ Nanoluc-sec virus, and new carbazole alkaloid **1** exhibited anti-HIV activity with an EC_50_ value of 2.4 μg/mL and SI of 7.1.

## 1. Introduction

**Figure 1:**
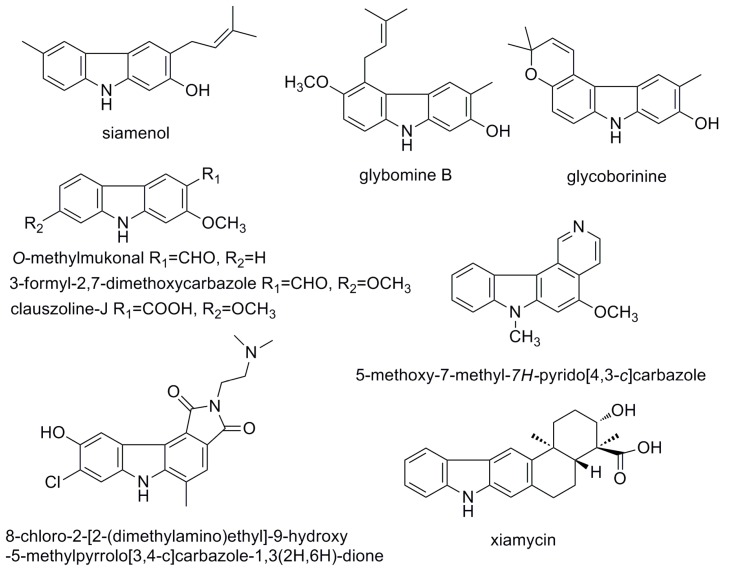
Natural and synthetic carbazole alkaloids with anti-HIV activity.

Carbazole alkaloids are characterized by a tricyclic aromatic basic skeleton with a central pyrrole ring fused between two benzene rings. The intriguing structural features and promising pharmacological activities of these natural products have led to enormous developments in the field of carbazole alkaloids [1,2]. Indeed, carbazoles have been widely investigated for their various biological properties, ranging from significant antimicrobial, antiprotozoal, and insecticidal effects to anti-inflammatory, antioxidative, antiplatelet aggregative, antiviral, and neuroprotective activities [1,2,3]. Natural and synthetic carbazole alkaloids have been reported to possess anti-HIV activity. Meragelman and co-workers found that an organic extract of *Murraya siamensis* from Thailand showed anti-HIV activity. Bioassay-guided fractionation of the extract led to the isolation of a C-6-prenylated carbazole alkaloid, siamenol, which exhibited HIV-inhibitory activity with an EC_50_ value of 2.6 µg/mL, reaching 50–60% maximum protection in the XTT-tetrazolium assay [4]. A crude extract from the underground parts of *Clausena excavata* inhibited HIV-1 activity in a syncytium assay [5]. Three carbazole alkaloids, *O*-methylmukonal, 3-formyl-2,7-dimethoxycarbazole, and clauszoline-J, displayed promising anti-HIV-1 effects with EC_50_ values of 2.7, 7.4, and 8.2 µg/mL, respectively, and potential therapeutic index (PTI) values of 56.7, 8.0, and 1.6, respectively [5]. The biological results supported the use of the *C. excavata* extracts for the treatment of AIDS infections [6]. In addition, the two C-5-prenylated carbazoles, glybomine B and glycoborinine, isolated from *Glycosmis montana* exhibited weak to moderate in vitro inhibitory activity against HIV replication in C8166 cells, with IC_50_ values of 9.73 and 4.47 µg/mL, respectively [7]. Hirata et al. prepared a series of *N*-alkyl-pyrido [4,3-*c*]carbazole derivatives starting from mukonal (2-hydroxy-9*H*-carbazole-3-carbaldehyde), a natural carbazole present in Rutaceous plants. Several compounds inhibited HIV replication in H9 lymphocytes. Among them, 5-methoxy-7-methyl-*7H*-pyrido[4,3-*c*]carbazole had the highest therapeutic index (TI = 503) in the series, with an EC_50_ value of 0.0054 µg/mL [3,8]. Yan et al. studied the antiviral activity of small molecular weight compounds with a carbazole structure using a single replication infectivity assay in HeLa 4.5/EGFP cells. 8-Chloro-2-[2-(dimethylamino)ethyl]-9-hydroxy-5-methylpyrrolo[3,4-c]carbazole-1,3(2*H*,6*H*)-dionedemonstrated potent strand-transfer inhibitory activity and could be used as a lead compound to develop novel inhibitors [9]. Later, a pentacyclic indolocarbazole, xiamycin, from *Streptomyces* sp. GT2002/1503, was found to exhibit selective anti-HIV activity (Figure 1) [10]. Based on the above results, carbazole may be considered a “privileged scaffold” that can provide opportunities for the development of anti-HIV drugs; however, this approach is a relatively low-studied area of anti-HIV drug discovery. Therefore, more multidirectional approaches need to be continued in anti-HIV drug discovery, including finding new natural products, evaluating known natural products, modifying semi-synthetic anti-HIV natural products, etc. [3,11].

The genus *Clausena* (Rutaceae) includes about 30 species, which are widely distributed in tropical and subtropical regions of the eastern hemisphere. About 10 species and 2 varieties are found in China, from the southwest to Taiwan [12]. Some species of this genus have been used in Asian folk medicine to treat various diseases for a long time [12]. Carbazole alkaloids are an important and characteristic chemotaxonomic feature typical of the genus *Clausena*. These compounds continue to attract high interest due to their structural diversity and promising pharmacological potential, i.e., antitumor, anti-inflammatory, antimicrobial, antifungal, anti-HIV, and neuroprotective activities [5,6,13,14,15,16,17,18,19,20,21,22,23]. *Clausena anisum-olens* Merr. is a perennial evergreen shrub or small tree mainly distributed in the Philippines, South China, and Southeast Asia. The aerial parts of *C. anisum-olens*, which grows in Yunnan Province, China, are used in folk medicine to treat dysentery and arthritis [12]. Our previous phytochemical investigations of this species led to the discovery of new monoterpenoid coumarins [12,24,25,26,27,28]. As part of our search for new natural products and bioactive compounds from medicinal plants of *Clausena* in Yunnan, a chemical reinvestigation of the ethanolic extract of the aerial parts of *C. anisum-olens* was then carried out. Two new carbazole alkaloids (**1**,**2**) together with six known carbazole alkaloids (**3**–**8**) were obtained. This paper describes the isolation, structural elucidation, and biological evaluation of the isolated carbazole alkaloids against HIV-1, using AZT as the standard in antiviral assays.

## 2. Results and Discussion

### 2.1. Structural Elucidation of the Compounds

**Figure 2:**
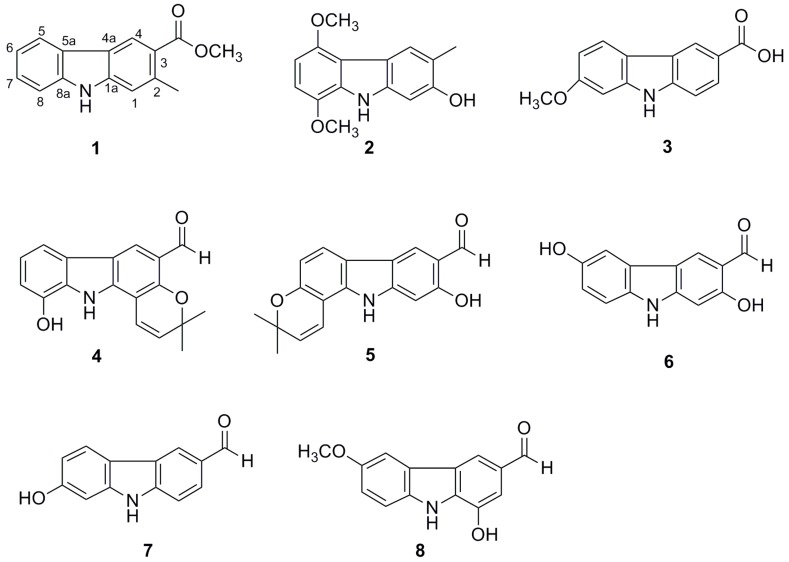
Structures of compounds **1**–**8**.

The 90% EtOH extract of the stems and leaves of *C. anisum-olen* Merr. was suspended in water and extracted successively with petroleum ether and ethyl acetate. The ethyl acetate fraction was repeatedly subjected to silica gel, Sephadex LH-20, RP-18 gel column chromatography to yield carbazole alkaloids **1**–**8**, including two new compounds, as shown in Figure 2.

Compound **1** was isolated as a white solid. The molecular formula, C_15_H_13_NO_2_, was established from^13^C NMR and HRESIMS data with 262.0839 [M + Na]^+^, calculated for 262.0838), suggesting 10 indices of hydrogen deficiency. The IR absorptions at 3354, 2946, 1692, 1634, 1488, 1452, 1381, and 1242 cm^−1^ showed the presence of a NH functionality from the carbazole alkaloid, methyl, ester carbonyl, and aromatic groups. The UV spectrum showed absorbances at 265 nm. Based on the IR and UV data, compound **1** was concluded to be a carbazole alkaloid [29,30].

**Figure 3:**
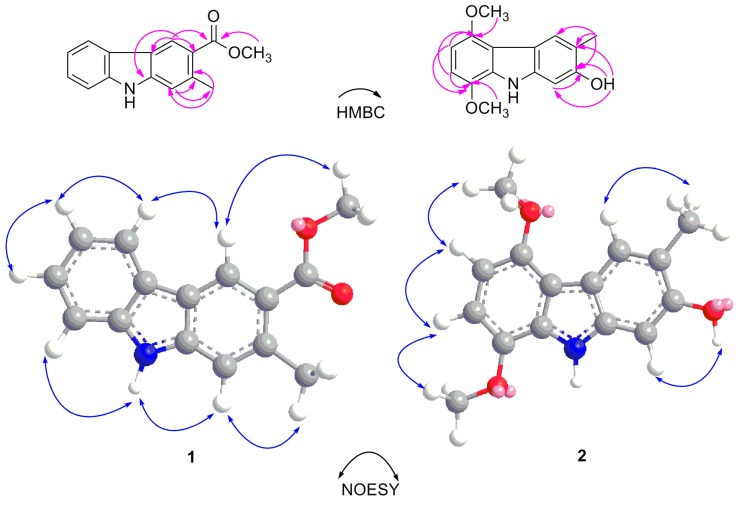
Key HMBC and NOESY correlations of **1** and **2**.

The ^1^H NMR spectrum of **1** (Table 1) indicated a set of *ortho*-disubstituted phenyl protons [δ_H_ 8.07 (1H, d, *J* = 7.8 Hz, H-5), 7.26 (1H, m, H-6), 7.42 (1H, m, overlap, H-7), 7.41 (1H, m, overlap, H-8); two lone aromatic protons [δ_H_7.22 (1H, s, H-1), 8.74 (1H, s, H-4)] in the aromatic region; a broad NH singlet at δ_H_ 8.15; a singlet for one aromatic methyl group at δ_H_ 2.77; and a methoxy group at δ_H_ 3.95. The ^13^C NMR spectrum (Table 1), classified by the HSQC spectrum, revealed the presence of 15 carbon atoms, including 12 aromatic carbons (6 sp^2^ carbon atoms and 6 sp^2^ quaternary carbon atom), an ester carbonyl, a methyl, and a methoxy. The NMR data implied that compound **1** was a carbazole alkaloid [29,30]. In addition, the 12 sp^2^ carbon atoms and 10 indices of hydrogen deficiency were attributable to one carbazole ring group and an ester carbonyl group. The HMBC spectrum provided valuable information about the structure of **1** (Figure 2). Thus, while the two- and three-bond correlations of the methine proton singlets at δ_H_ 7.22 (H-1) and δ_H_ 8.74 (H-4), as well as the aromatic methyl proton signal at δ_H_ 3.95 with those of the neighboring carbons, clearly suggested the substitution at positions 2 and 3 in the molecule. The HMBC correlations from H-4 to the ester carbonyl carbon (δ_C_ 168.5) located the ester carbonyl at C-3 (δ_C_ 138.8). Additionally, correlations from the methyl proton to C-1 (δ_C_ 112.8), C-2 (δ_C_ 121.2), and C-3 (δ_C_ 138.8) supported that the location of the aromatic methyl group at C-2. The structure of **1** was finally confirmed by the clear NOE interactions observed in its NOESY spectrum, as depicted in Figure 3 (see Supplementary Materials). Hence, the complete structure of **1** (clauolenzole A (2-methyl-9*H*-carbazole-3-methyl carboxylate)) was established as shown.

Compound **2** was obtained as a brown-yellow solid. The HRESIMS gave a molecular ion peak at *m*/*z* 256.0981 [M − H]^−^ (calculated 256.0979) consistent with the molecular formula C_15_H_15_NO_3_, implying nine degrees of unsaturation. The UV spectrum showed absorbances at 234 nm. The IR spectrum displayed absorptions characteristic of hydroxy (3442 cm^−1^), aromatic ring (1600, 1521, 1452 cm^−1^), and methyl (2927, 2852, 1401, 1360 cm^−1^) groups. Thus, compound **2** was defined as having a carbazole alkaloid skeleton.

The ^1^H NMR spectrum of **2** showed the presence of three singlet protons, one for a phenolic hydroxyl group at δ_H_ 8.09 (1H, s, OH-2) and the other two for aromatic methine protons at δ_H_ 7.87 (1H, s, H-4) and 6.97 (1H, s, H-1), as well as an *ortho*-coupled aromatic proton [δ_H_ 6.69 (1H, d, *J* = 5.6 Hz, H-7) and 6.46 (1H, d, *J* = 5.6 Hz, H-6)] and a broad NH singlet at δ_H_ 9.87 (1H, brs, NH) in the aromatic proton region. The ^1^H NMR spectrum also displayed a single methyl peak at δ_H_ 2.29 (3H, s, 3-CH_3_) and two methoxy peaks at δ_H_ 3.93 (3H, s) and 3.86 (3H, s) at high field. These assignments are consistent with the ^13^C NMR data of **2** (Table 1), with resonances for 15 carbons: 12 aromatic carbons from two benzene rings, one methyl carbon, and two methoxy carbons. The long-range correlations in the HMBC experiment were observed between the methoxy groups (δ_H_ 3.93 and 3.86) and C-5 (δ_C_ 149.8 s) and C-8 (δ_C_ 140.4 s), respectively (Figure 3). Two methoxy (5-OCH_3_ and 8-OCH_3_) positions were further confirmed from the HMBC correlations between H-6 (δ_H_ 6.46) and C-5 (δ_C_ 149.8 s) and C-8 (δ_C_ 140.4 s), between H-7 (δ_H_ 6.69) and C-5 (δ_C_ 149.8 s) and C-8 (δ_C_ 140.4 s), as well as NOESY correlations between H-6 (δ_H_ 6.46) and 5-OCH_3_ (δ_H_ 3.93), between H-7 (δ_H_ 6.69) and 8-OCH_3_ (δ_H_ 3.86), and between H-6 (δ_H_ 6.46) and H-7 (δ_H_ 6.69). In addition, the HMBC data revealed three- and two-bond correlations between the phenolic hydroxyl group at δ_H_ 8.09 and C-1 (δ_C_ 96.2 d), C-2 (δ_C_ 153.9 s), and C-3 (δ_C_ 116.8 s) and between the aromatic methyl group at δ_H_ 2.29 and C-2 (δ_C_ 153.9 s), C-3 (δ_C_ 116.8 s), and C-4 (δ_C_ 123.8 d). The hydroxyl (2-OH) and methyl (3-CH_3_) positions were confirmed by the correlations between H-1 (δ_H_ 6.97) and OH-2 (δ_H_ 8.09) and between H-4 (δ_H_ 7.87) and CH_3_-3 (δ_H_ 2.29) in the NOESY spectrum of **2** (Figure 3). Ultimately, compound **2** was determined to be 5,8-dimethoxy-3-methyl-9H-carbazol-2-ol, named clauolenzole B.

The six known carbazole alkaloids were identified as clausine N (**3**) [31], heptazolicine (**4**) [32], clauszoline B (**5**) [33], clausine-O (**6**) [31], 7-dihydroxy-9*H*-carbazole-3-carboxaldehyde (**7**) [34], and clausine I (**8**) [35], based on their spectroscopic profiles and comparison with literature values.

### 2.2. Evaluation of In Vitro Anti-HIV Activity

All eight carbazole alkaloids isolated from *C. anisum-olens* were evaluated for anti-HIV activity by determining the inhibitory effects on virus replication in MT-4 lymphocytes infected by HIV-1_NL4-3_ Nanoluc-sec virus. Table 2 shows the anti-HIV results for the tested compounds with azidothymidine (AZT) as a positive control. New carbazole alkaloid **1** exhibited an anti-HIV EC_50_ value of 2.4 μg/mL and SI of 7.1. Thus, this compound might be a useful compound to further study carbazole alkaloids as potential anti-HIV agents. The remaining seven compounds did not show selective activity (CC_50_/EC_50_ < 5). The results prompted us to pursue whether synthetic modifications of the carbazole alkaloid structure could increase the anti-HIV-1 activity.

## 3. Materials and Methods 

### 3.1. General Experimental Procedures 

UV spectra were measured on a Shimadzu UV-2401PC spectrophotometer (Shimadzu Co. Ltd., Tokyo, Japan). IR spectra were obtained on a Bio-Rad FTS-135 (Bio-Rad, Richmond, Canada) infrared spectrophotometer. The 1D and 2D NMR spectra were obtained at 400 and 100, and 600 and 150 MHz for ^1^H and ^13^C, respectively, on Bruker DRX-400 and 600 spectrometers (Bruker, Bremerhaven, Germany) with TMS as an internal standard. MS data were recorded on a VG Autospec-3000 mass spectrometer (VG, Manchester, England). Commercially available silica gel (100−200 mesh or 200−300 mesh, Qingdao Makall Chemical Co. Ltd., Qingdao, China), Lobar LiChroprep RP-18 (40−63 μm, Merck, St. Louis, MO, USA), and Sephadex LH-20 (Pharmacia, Fine Chemical Co. Ltd., Sweden) were used for open-column chromatography. All solvents were distilled prior to use.

### 3.2. Plant Material 

The leaves and twigs of *Clausena anisum-olens* Merr. were collected in the Hekou County of Yunnan Province, People’s Republic of China, in May 2015 and identified by Professor Yu Chen of the Kunming Institute of Botany. A voucher specimen (No. 02041705) was deposited in the State Key Laboratory of Phytochemistry and Plant Resources in West China, Kunming Institute of Botany, Chinese Academy of Sciences.

### 3.3. Extraction and Isolation

The powdered leaves and twigs of *Clausena anisum-olens* Merr. (5.0 kg) were repeatedly extracted with 90% aqueous EtOH (3 × 10 L) at room temperature. The extract was concentrated under reduced pressure to give a brown syrup, which was partitioned into H_2_O and extracted successively with petroleum ether and ethyl acetate; the extracts were kept separately. The ethyl acetate extract (50.5 g) was subjected to silica gel column chromatography, eluting with petroleum ether-EtOAc (4:1, 2:1, 1:1, 2:3), EtOAc, EtOAc–MeOH (8:2, 7:3, 6:4, 1:1), and finally MeOH to afford eight fractions (I–VIII). Fraction II (12 g) was subjected again to silica gel column chromatography on Pharmadex LH-20 (MeOH) to give compound **1** (5 mg). Fraction III (6.2 g) was re-subjected to silica gel column chromatography on Pharmadex LH-20 (MeOH) to afford compounds **2** (3 mg) and **8** (3 mg). Fraction IV (14.2 g) was re-subjected to silica gel column chromatography on Pharmadex LH-20 (MeOH) and RP C18 to afford compounds **4** (3 mg) and **5** (6 mg). Fraction V (8 g) was subjected again to silica gel column chromatography on Pharmadex LH-20 (MeOH) to give compounds **3** (15 mg), **6** (6 mg), and **7** (8 mg).

*Clauolenzole A* (**1**).White solid. UV (MeOH) λ_max_ 265 nm; IR (KBr) ν_max_ 3354, 2946, 1692, 1634, 1488, 1452, 1381, 1242 cm^−1^; ^1^H and ^13^C NMR data see Table 1; HRESIMS [M + Na]^+^ *z*/*z* 262.0839 (calculated for C_15_H_13_NO_2_, 262.0838).

*Clauolenzole B* (**2**). Ochre solid. UV (MeOH) λ_max_ 234 nm; IR (KBr) ν_max_ 3442, 2927, 2852, 1600, 1521, 1452, 1401, 1360 cm^−1^; ^1^H and ^13^C NMR data see Table 1; HRESIMS [M − H]^−^ *z*/*z* 256.0981 (calculated for C_15_H_15_NO_3_, 256.0979).

### 3.4. Bioassay Methods

MT4 cells (1 × 10^5^ cells mL^−1^) in 96 well plates were infected by HIV-1 NL4-3 Nanoluc-sec at a dose of 50 TCID_50_/well in the presence of each test compound at various concentrations. On Day 3 post-infection, supernatants were collected and assayed for luciferase activity using the Nano-Glo Luciferase Assay System (Promega, Madison, WI, USA). The antiviral potency was defined as the compound concentration that reduces the luciferase activity by 50% (EC_50_) [36].

The cytotoxicity of the compound against MT4 cells was assessed using a CytoTox-Glo cytotoxicity assay (Promega, Madison, WI, USA). MT-4 cells were cultured in the presence of various concentrations of each test compound for 3 days, and the 50% cytotoxic concentration (CC_50_) causing a 50% reduction of cell viability was determined by following the manufacturer’s protocol [36].

## 4. Conclusions

In this work, eight carbazole alkaloids (**1**–**8**) were isolated and identified based on spectroscopic analyses and references from *C. anisum-olens*. Among them, compounds **1** and **2** were two new carbazole alkaloids, named clauolenzole A and B, respectively. Meanwhile, compounds **1**–**8** were evaluated for their in vitro anti-HIV activity on virus replication in MT-4 lymphocytes infected by HIV-1_NL4-3_ Nanoluc-sec virus with azidothymidine (AZT) as positive control. The new carbazole alkaloid **1** exhibited an anti-HIV EC_50_ value of 2.4 μg/mL and SI of 7.1, comparable or better than the values for similar carbazoles. Based on the current and prior literature results [4,5,6,7,8,9,10], carbazole alkaloids might be candidates for further study as potential anti-HIV agents. Structural analyses and synthetic modification of carbazole alkaloids and their derivatives are in progress to possibly increase the compounds’ anti-HIV-1 activity.

## Figures and Tables

**Table 1:** 600MHz and ^13^C NMR (150MHz) data of clauolenzoles A and B (**1**,**2**).

Position	1 (CDCl_3_)		2 (acetone-*d*_6_)	
	δ_H_ (*J* in Hz)	δ_C_, Type	δ_H_ (*J* in Hz)	δ_C_, Type
1	7.22, s	112.8, CH	6.97, s	96.2, CH
2	/	121.2, C	/	153.9, C
3	/	138.8, C	/	116.8, C
4	8.74, s	124.1, CH	7.87, s	123.8, CH
5	8.07, d (7.8)	120.4, CH	/	149.8, C
6	7.26, m	120.2, CH	6.46, d (5.6)	98.8, CH
7	7.42, m, overlap	126.1, CH	6.69, d (5.6)	104.8, CH
8	7.41, m, overlap	110.8, CH	/	140.4, C
1a	/	141.9, C	/	139.2, C
4a	/	123.4, C	/	116.0, C
5a	/	121.1, C	/	113.8, C
8a	/	139.9, C	/	130.8, C
2-CH_3_	2.77, s	23.0, CH_3_		
3-CH_3_			2.29, s	15.8, CH_3_
3-COOCH_3_	/	168.5, C		
	3.95, s	51.6, CH_3_		
5-OCH_3_			3.93, s	54.9, CH_3_
8-OCH_3_			3.86, s	55.3, CH_3_
2-OH			8.09, s	/
NH	8.15, br s	/	9.87, br s	/

**Table 2:** In vitro anti-HIV data of compounds **1**–**8** ^a^.

Compound	EC_50_ (μg/mL) NL4-3 ^b^	CC_50_ (μg/mL) MT4 ^c^	TI ^e^
**1**	2.4 ± 0.53	17 ± 2.5	7.1
**2**	*- ^d^ 3.7 ± 1.9	15.2 ± 3.1	
**3**	*- ^d^		
**4**	*- ^d^		
**5**	*- ^d^		
**6**	*- ^d^		
**7**	>25	>25	
**8**	*- ^d^		
**AZT** ^f^	0.0055 ± 0.0018	>0.1	18.2

^a^ The highest concentrations for the tested compounds and AZT were 25 μg/mL and 0.1 μg/mL, respectively. Testing was done with a series of 4-fold dilutions (six concentrations). ^b^ EC_50_: 50% HIV-inhibitory concentration (mean ± SD of 3 tests). ^c^ CC_50_: 50% cytotoxic concentration. ^d^ *-: no selective anti-HIV activity (CC_50_/EC_50_ < 5). ^e^ TI: CC_50_/EC_50_. ^f^ AZT: azidothymidine.

## Supplementary Materials

The following are available online: ^1^H, ^13^C, 2D NMR, and HRMS spectra for **1**–**2**.
