# Supplementary material for: Carbazole Alkaloids from Clausena anisum-olens: Isolation, Characterization, and Anti-HIV Evaluation
Source: Molecules. 2019 Dec 26;25(1):99. doi: 10.3390/molecules25010099 (PMC6983056; doi:10.3390/molecules25010099)
Supplement: Supplementary file 1 [file molecules-25-00099-s001.pdf]

Carbazole alkaloids from *Clausena anisum-olens*: isolation, characterization and anti-HIV evaluation

Jing-Hua Yang[1,†], Xin-Yi Wang[2,†], Yi-Ping Zhou[3], Rong Lu[1], Chin-Ho Chen[4], Meng-Han Zhang[5], Yung-Yi Cheng[5,6], Susan L. Morris-Natschke[5], Kuo-Hsiung Lee[5,6,*] and Yun-Song Wang[1,*]

[1] Key Laboratory of Medicinal Chemistry for Natural Resource, Ministry of Education, School of Chemical Science and Technology, Yunnan University, Kunming 650091, China
[2] The High School Affiliated to Yunnan Normal University, Kunming 650106, China
[3] School of Pharmaceutical Sciences & Yunnan Key Laboratory of Pharmacology for Natural Products, Kunming Medical University, Kunming 650500, China
[4] Surgical Science, Department of Surgery, Duke University Medical Center, Durham, NC, 27710, USA
[5] Natural Products Research Laboratories, UNC Eshelman School of Pharmacy, University of North Carolina, Chapel Hill, NC, 27599, USA
[6] Chinese Medicine Research and Development Center, China Medical University and Hospital, Taichung 40402, Taiwan

List of Figures

Figure S1. ^{1}H NMR spectrum of **1** in $CDCl_3$

Figure S2. ^{13}C NMR and DEPT spectra of **1** in $CDCl_3$

Figure S3. HSQC spectrum of **1** in $CDCl_3$

Figure S4. HMBC spectrum of **1** in $CDCl_3$

Figure S5. ^{1}H-^{1}H COSY spectrum of **1** in $CDCl_3$

Figure S6. ROESY spectrum of **1** in $CDCl_3$

Figure S7. HRESIMS spectrum of **1**

Figure S8. ^{1}H NMR spectrum of **2** in acetone-d_6

Figure S9. ^{13}C NMR and DEPT spectra of **2** in acetone-d_6

Figure S10. HSQC spectrum of **2** in acetone-d_6

Figure S11. HMBC spectrum of **2** in acetone-d_6

Figure S12. ^{1}H-^{1}H COSY spectrum of **2** in acetone-d_6

Figure S13. ROESY spectrum of **2** in acetone-d_6

Figure S14. HRESIMS spectrum of **2**

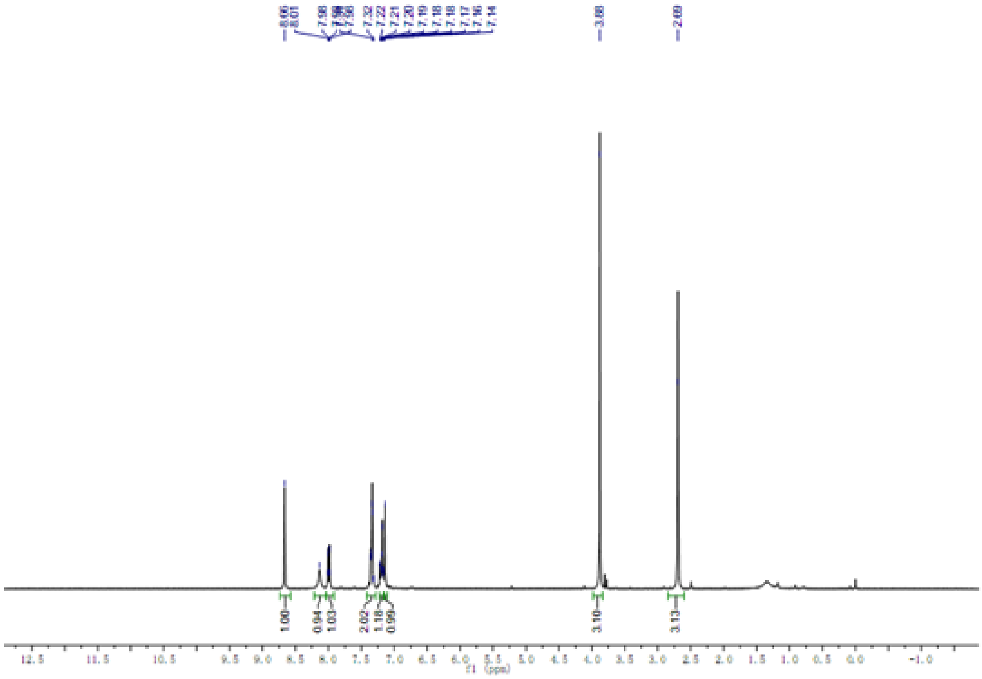

Figure S1. ^{1}H NMR spectrum of **1** in $CDCl_3$

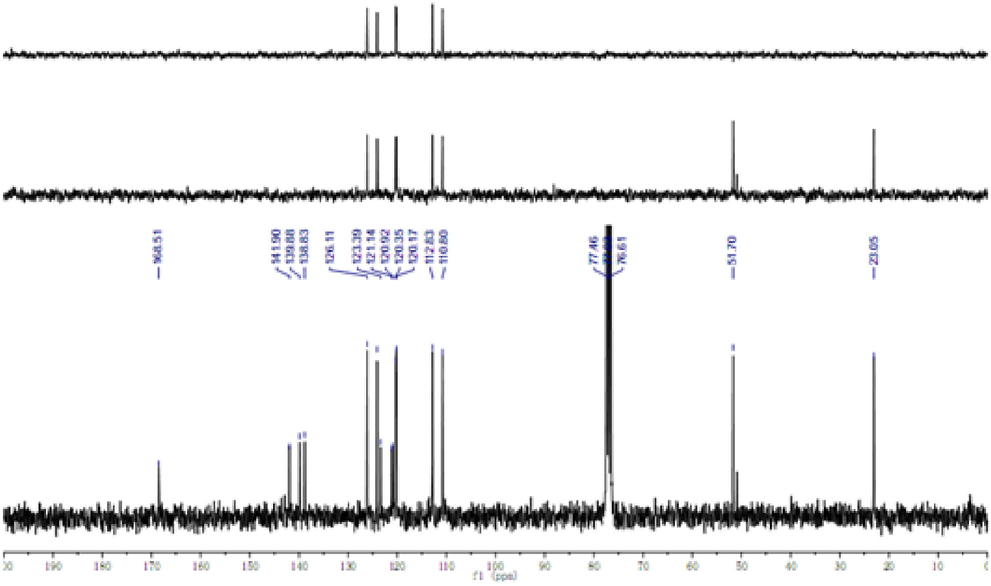

Figure S2. ^{13}C NMR and DEPT spectra of **1** in $CDCl_3$

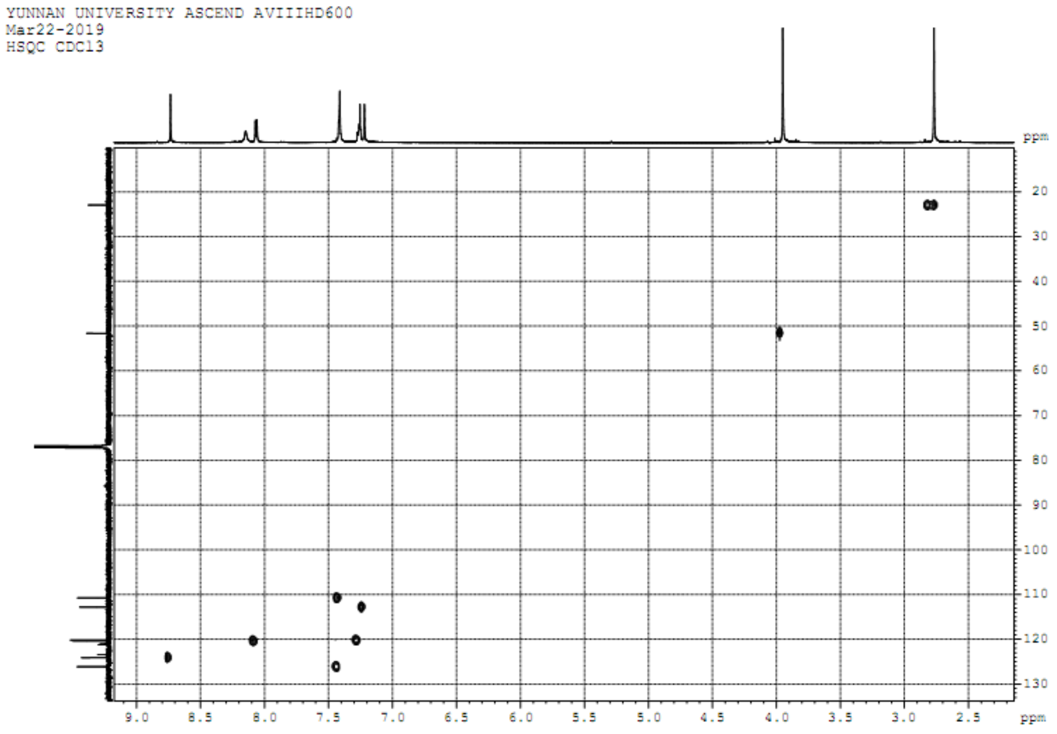

Figure S3. HSQC spectrum of **1** in $CDCl_3$

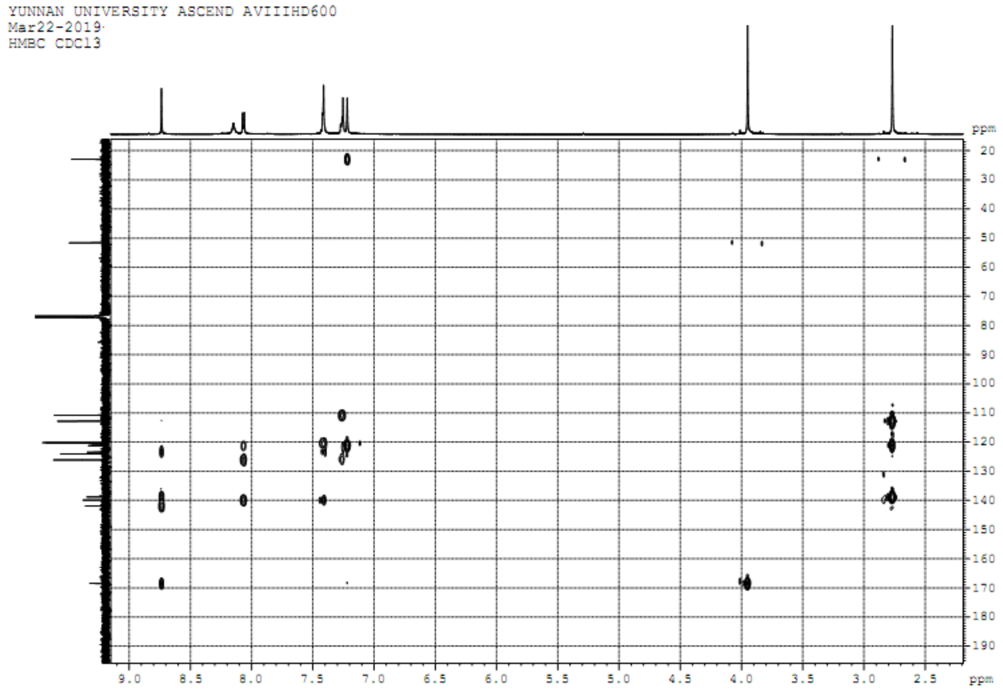

Figure S4. HMBC spectrum of **1** in $CDCl_3$

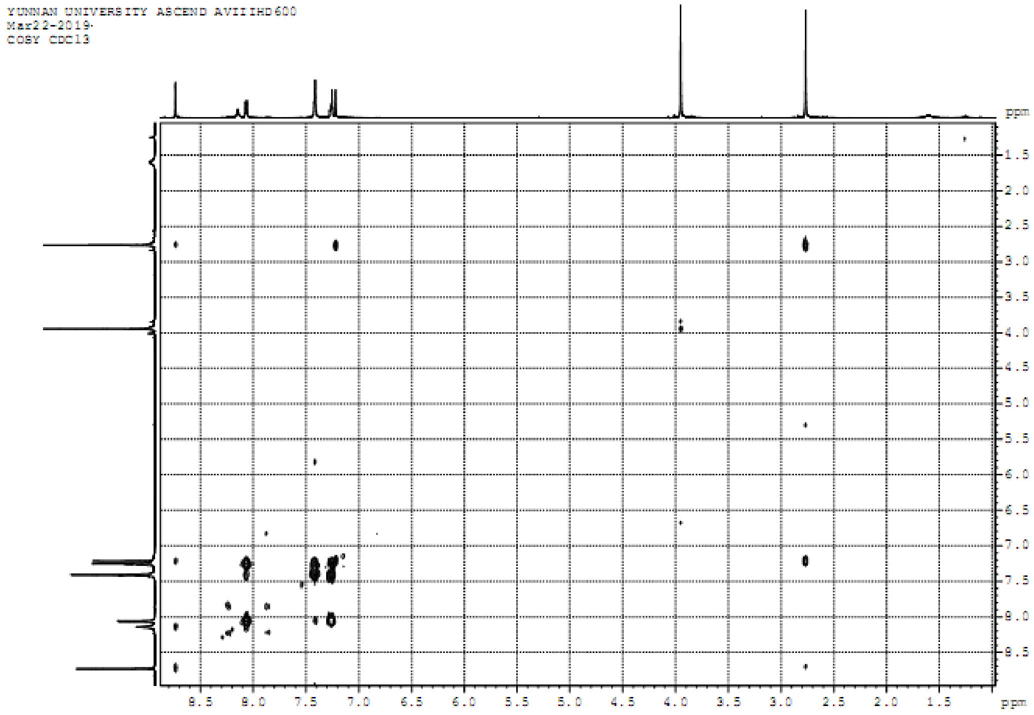

Figure S5. ^{1}H-^{1}H COSY spectrum of **1** in $CDCl_3$

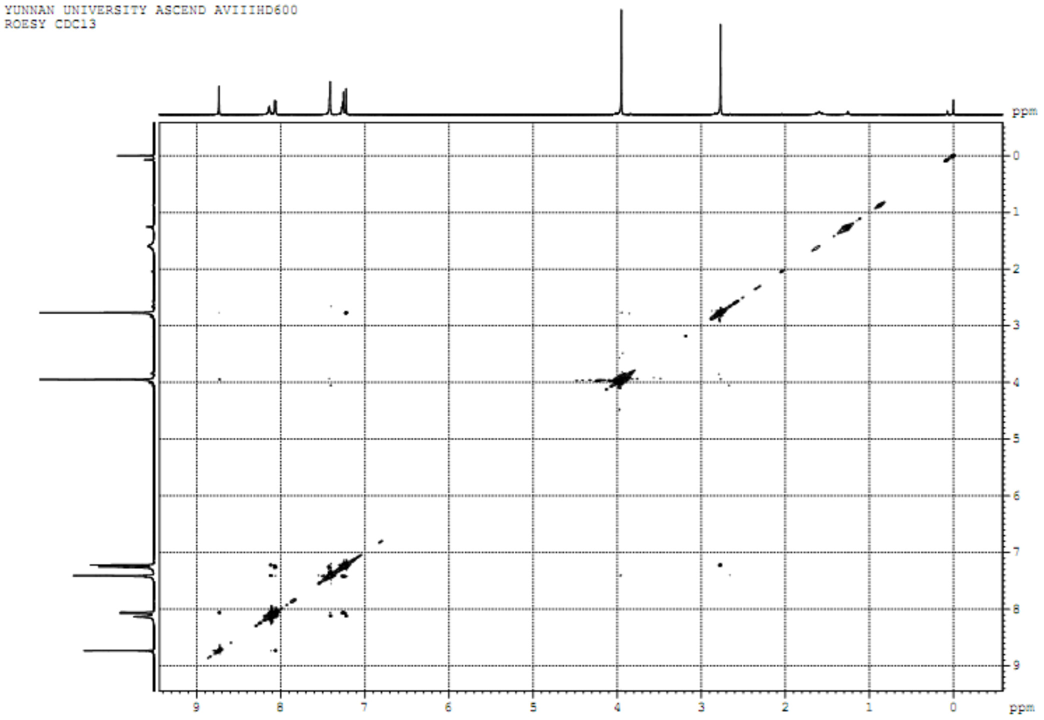

Figure S6. ROESY spectrum of **1** in $CDCl_3$

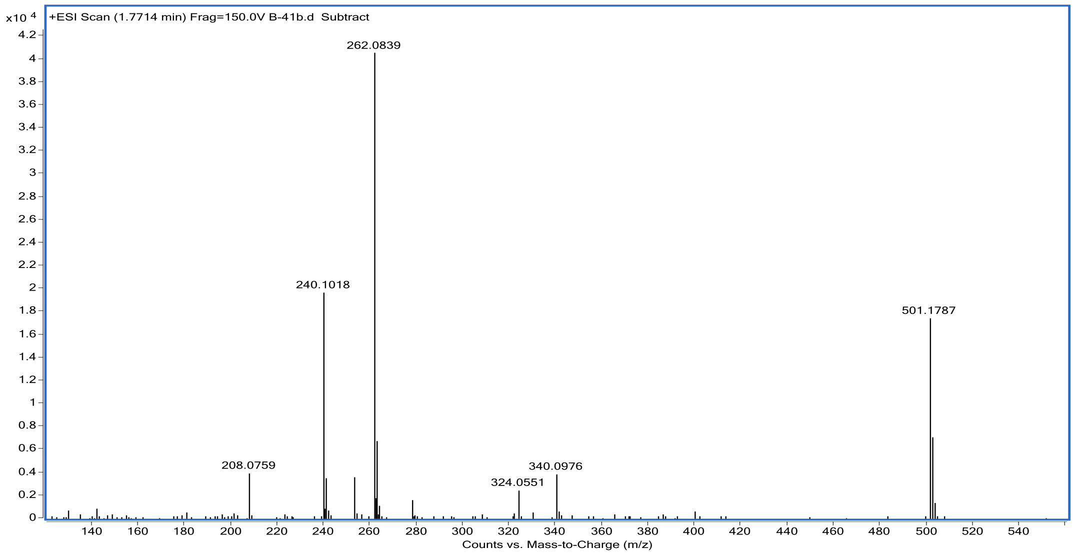

Figure S7. HRESIMS spectrum of **1**

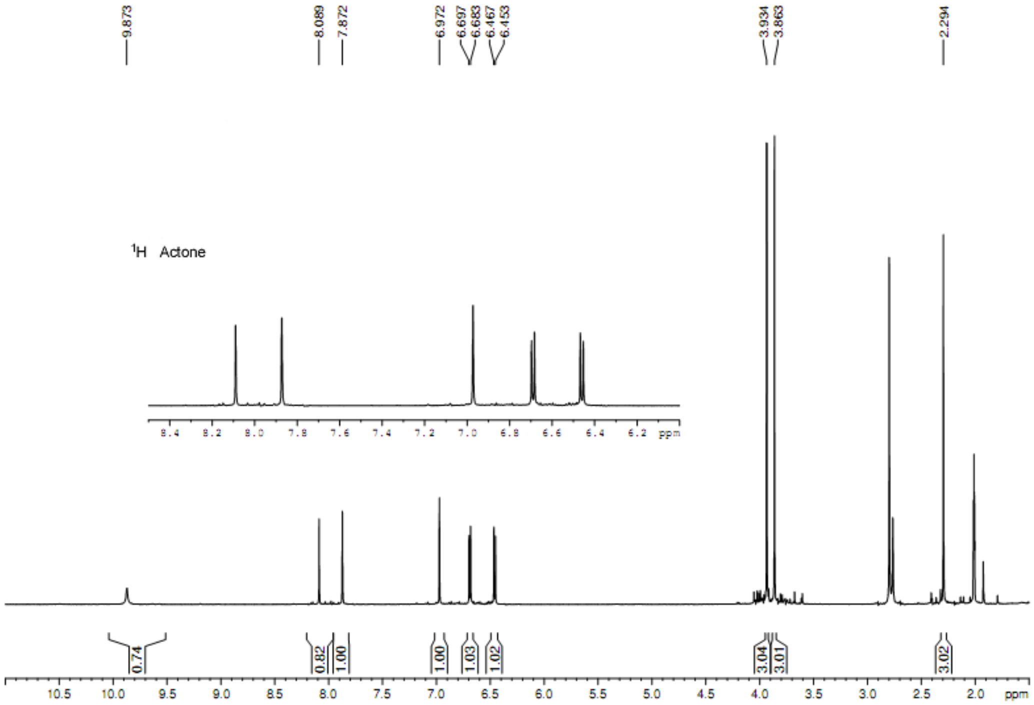

Figure S8. ^{1}H NMR spectrum of **2** in acetone-d_6

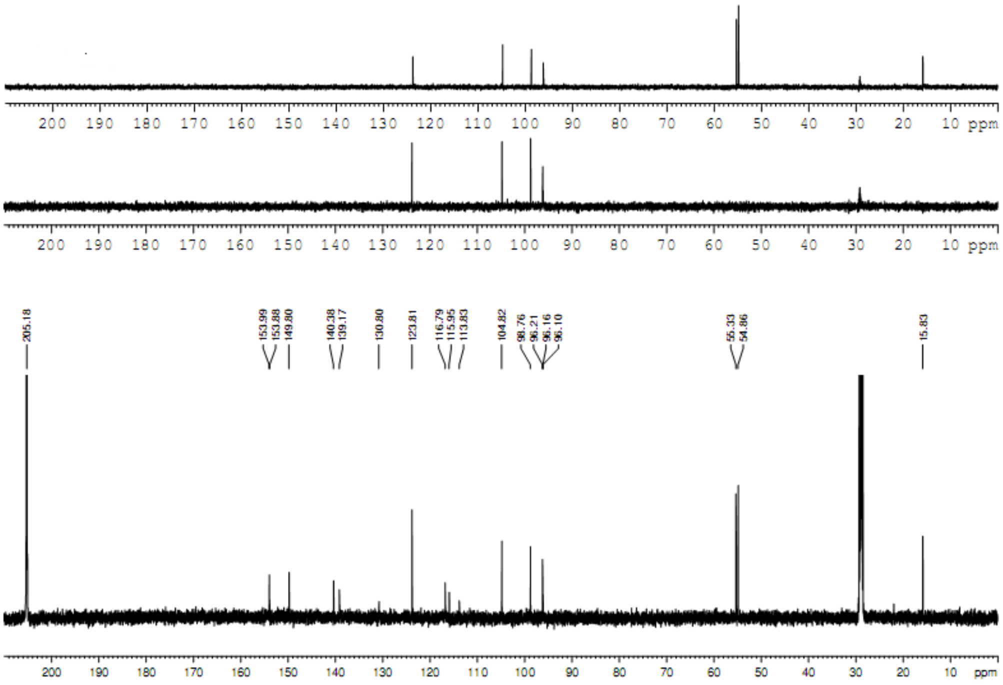

Figure S9. ^{13}C NMR and DEPT spectra of **2** in acetone-d_6

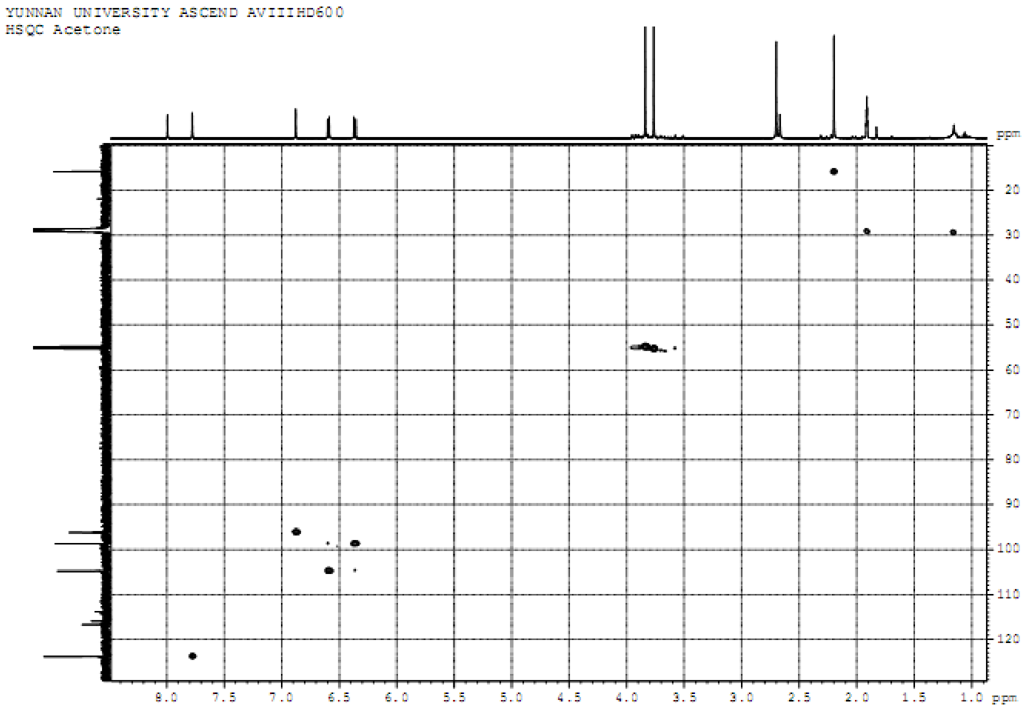

Figure S10. HSQC spectrum of **2** in acetone-d_6

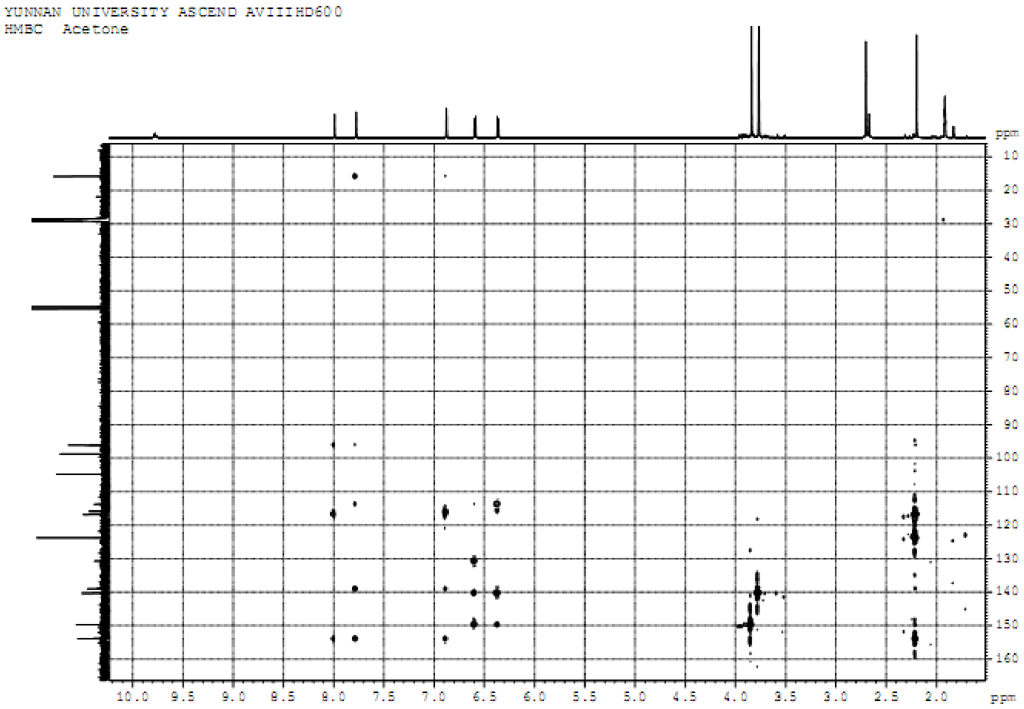

Figure S11. HMBC spectrum of **2** in acetone-d_6

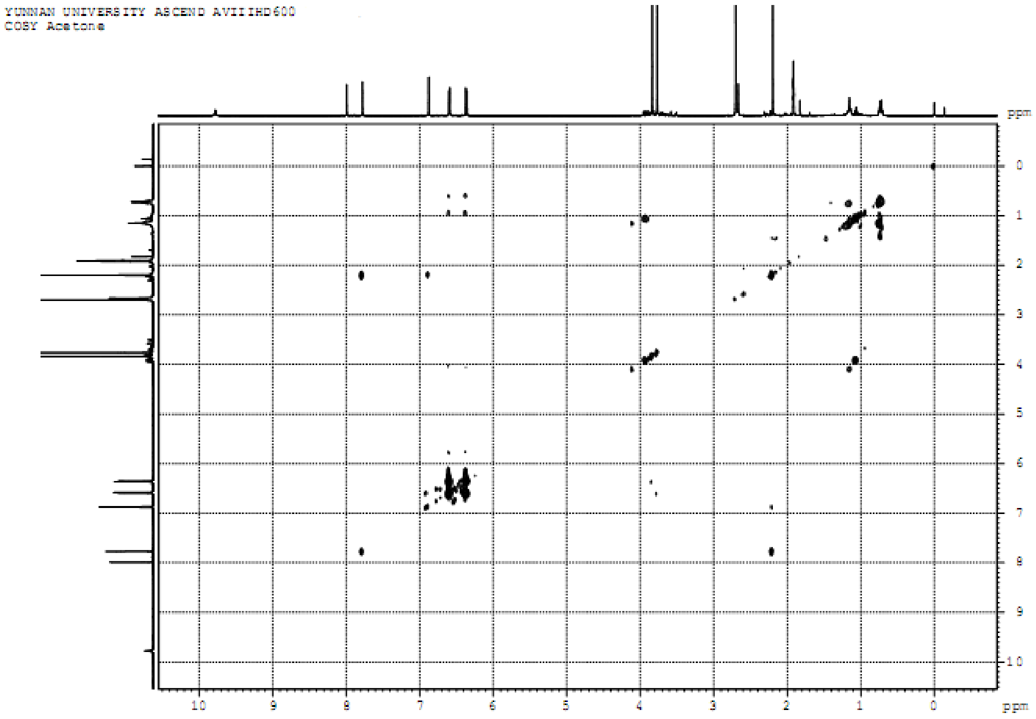

Figure S12. ^1H-^1H COSY spectrum of **2** in acetone-d_6

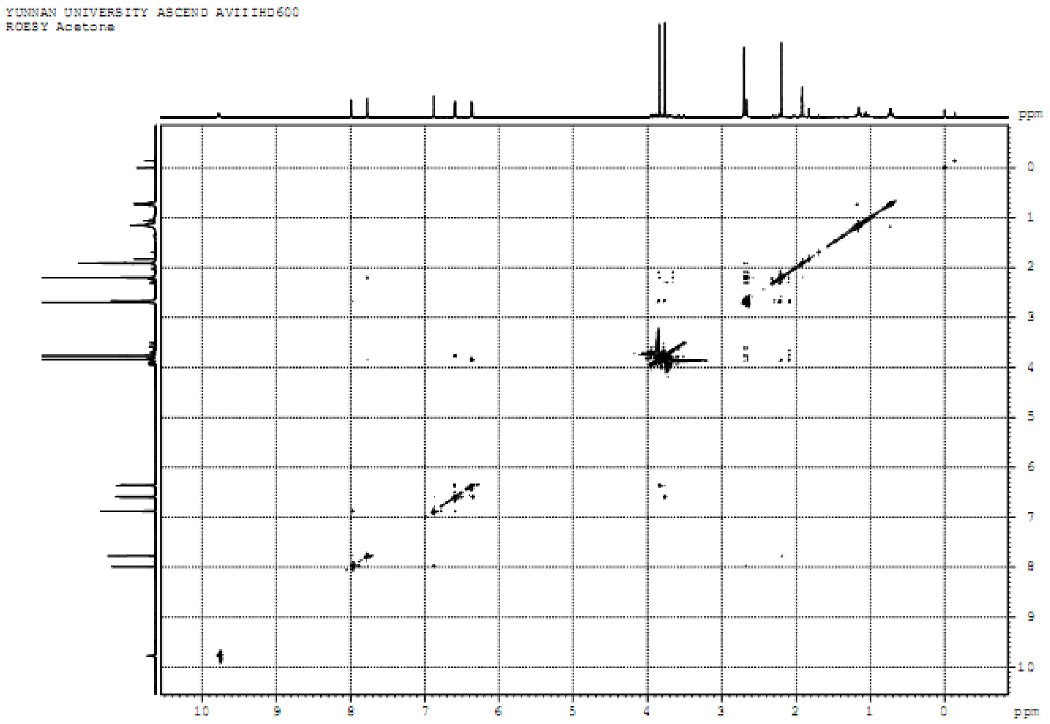

Figure S13. ROESY spectrum of **2** in acetone-d_6

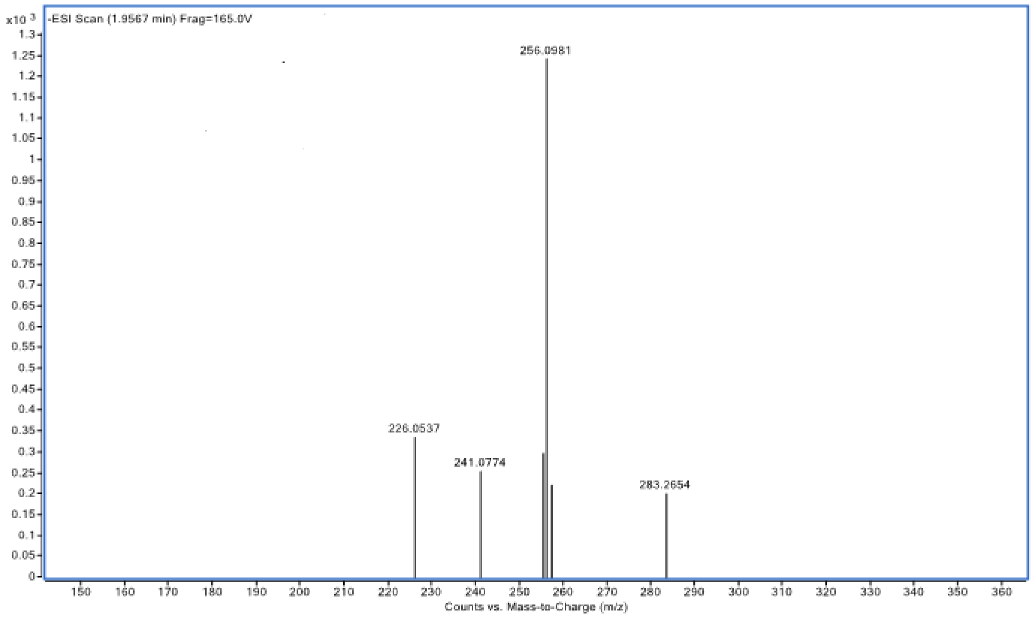

Figure S14. HRESIMS spectrum of **2**
